# Adipose Stem Cell Mitochondrial Transplantation in ART: From Biological Rationale to Clinical Milestone

**DOI:** 10.3390/cells15161438

**Published:** 2026-08-10

**Authors:** Helaruwan Pasan Kumara Wijethunga Arachchilage, Sanath Udayanga Kankanam Gamage, Atsushi Morimoto, Yoshiharu Morimoto

**Affiliations:** 1 Wish Fertility Hospital Pvt Ltd., 30 Elhena Rd, Maharagama 10230, Sri Lanka; helaruwanpasankumara@gmail.com; 2 HORAC Grand Front Osaka Clinic, 3−1 Ofukacho, Kita Ward, Osaka 530-0011, Japan; ats.japan.0930@gmail.com (A.M.); ivfceo@gmail.com (Y.M.)

**Keywords:** Adipose Stem Cell Energy Transfer (ASCENT), adipose-derived stem cells, mitochondrial transplantation, mitochondrial dysfunction, autologous therapy, Mito-ICSI

## Abstract

**Highlights:**

**What are the main findings?**

- Mitochondrial dysfunction is a central driver of poor oocyte competence in ART.
- Chemical support cannot restore mitochondrial mass once depletion is advanced.

**What are the implications of the main findings?**

- Autologous mitochondrial transfer (ASCENT) avoids the constraints of heterologous replacement.
- Preclinical efficacy and transgenerational safety support clinical evaluation in ART.

**Abstract:**

Oocyte quality is the primary determinant of success in assisted reproductive technologies (ART), and mitochondrial dysfunction is increasingly recognized as a central mediator of poor oocyte competence across advanced maternal age, recurrent implantation failure, polycystic ovary syndrome, endometriosis, and obesity. Chemical interventions improve the mitochondrial microenvironment but cannot restore depleted mitochondrial mass, while heterologous mitochondrial replacement remains constrained by ethical, legal, and biological limitations. This review examines the biological basis for mitochondrial intervention in oocytes, evaluates chemical and cellular therapeutic approaches, and assesses the evidence for autologous Adipose Stem Cell-derived Mitochondria ENergy Transfer (ASCENT). Mitochondria govern oocyte ATP production, calcium-mediated meiotic integrity, and redox homeostasis, and their disruption contributes to aneuploidy, fertilization failure, and embryonic arrest. Among cellular interventions, autologous adipose-derived stem cell mitochondrial transplantation offers minimally invasive tissue accessibility, morphological compatibility with oocyte mitochondria, robust membrane potential, and a preclinically validated Mito-ICSI delivery platform. Notably, ASCENT is currently the only autologous approach for which safety across three consecutive offspring generations has been reported in a mammalian model, with primary maternal origin of offspring mtDNA confirmed. Together, preclinical efficacy, transgenerational safety, and human proof-of-concept support progression toward a rigorously designed clinical trial, while ASC-derived mitochondria hold broader relevance in regenerative medicine.

## 1. Introduction

Infertility affects approximately one in six couples worldwide, and the demand for assisted reproductive technologies (ART) continues to grow steadily [1]. Despite remarkable advances in ovarian stimulation protocols, embryo culture systems, and cryopreservation techniques, the overall success rates of ART remain limited, with live birth rates per cycle still falling well below 50% in most clinical settings [2]. Among the many determinants of ART outcome, oocyte quality stands as the single most critical factor governing fertilization success, embryo development, and ultimately, the establishment of a healthy pregnancy [3].

Oocyte quality is a multidimensional concept encompassing nuclear maturation, cytoplasmic competence, and the integrity of the cellular machinery required to support fertilization and early embryogenesis [4]. Central to all of these processes is mitochondrial function. As the primary sites of ATP production through oxidative phosphorylation, mitochondria supply the energy demands of oocyte maturation, meiotic spindle assembly, chromosome segregation, fertilization, and the initial cleavage divisions of the early embryo [5,6]. Beyond energy production, mitochondria regulate calcium homeostasis, redox signaling, and apoptosis all of which are indispensable for oocyte developmental competence [7,8]. Given this multifaceted role, it is unsurprising that mitochondrial dysfunction has been consistently implicated in poor oocyte quality, recurrent implantation failure, and age-related reproductive decline [9,10].

The decline in mitochondrial quality and quantity within oocytes is particularly pronounced with advancing maternal age the very demographic that most frequently seeks ART treatment [11]. Conditions such as polycystic ovary syndrome (PCOS), obesity, endometriosis, and diabetes further compound mitochondrial dysfunction in oocytes, creating a broad clinical need for strategies that directly address mitochondrial insufficiency [12,13]. While antioxidant supplementation and lifestyle modifications have shown some benefit in improving the mitochondrial microenvironment, these approaches act indirectly and cannot restore the mitochondrial mass or function of a compromised oocyte [14].

Mitochondrial replacement therapies (MRT), such as maternal spindle transfer and pronuclear transfer, offer a more direct cellular solution but involve germline modification using donor mitochondrial DNA, raising substantial ethical, legal, and biological concerns that limit their widespread application [15]. Autologous mitochondrial transplantation in which a patient’s own healthy mitochondria are isolated from somatic or stem cells and transferred directly into her oocytes circumvents these concerns entirely. Among the candidate cell sources explored for this purpose, adipose-derived stem cells (ASCs) have emerged as particularly promising, owing to their accessibility, robust mitochondrial membrane potential, morphological compatibility with oocyte mitochondria, and established safety profile [16].

Building on preclinical evidence demonstrating improved oocyte developmental competence, enhanced embryo quality, and confirmed transgenerational safety, the Adipose Stem Cell-derived Mitochondria ENergy Transfer (ASCENT) approach represents a clinically translatable and ethically sound strategy for addressing mitochondrial dysfunction in ART [17,18]. This review examines the biological rationale for mitochondrial intervention in oocytes, evaluates current chemical and cellular therapeutic approaches, and presents the evidence supporting ASCENT as a meaningful milestone in the advancement of fertility treatment.

## 2. Mitochondrial Function as the Foundation of Oocyte Competence

**Figure 1:**
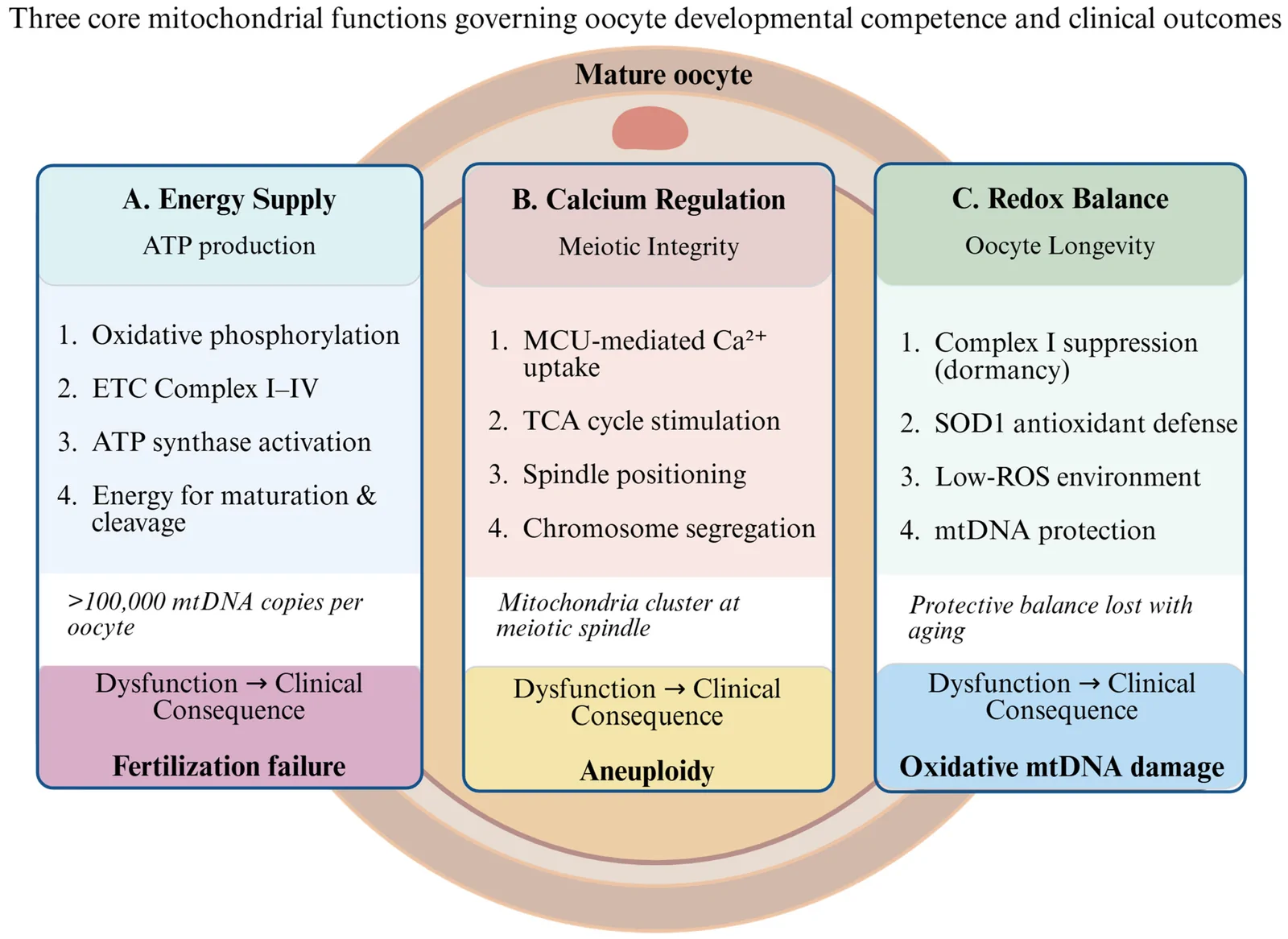
**The Mitochondrial Triad of Oocyte Competence**. Three-panel schematic of core mitochondrial functions in the oocyte: (**A**) adenosine triphosphate (ATP) production via oxidative phosphorylation (**B**) calcium regulation via the mitochondrial calcium uniporter (MCU) (**C**) redox balance and complex I suppression in dormant oocytes. Insets indicate the clinical consequence of dysfunction in each domain. ATP, adenosine triphosphate; MCU, mitochondrial calcium uniporter; mtDNA, mitochondrial DNA.

The oocyte is among the most metabolically demanding cells in the human body, and its developmental competence is inextricably linked to the number, quality, and functional integrity of its mitochondria [4]. Unlike most somatic cells, the oocyte cannot readily replenish its mitochondrial pool during the critical windows of maturation and early embryogenesis it must rely entirely on the mitochondrial reserves accumulated during folliculogenesis [19,20]. This unique biological constraint makes the oocyte exquisitely sensitive to any mitochondrial insufficiency. Understanding precisely how mitochondria support oocyte function is therefore essential for appreciating both the clinical consequences of their dysfunction and the therapeutic rationale for mitochondrial transplantation (Figure 1).

### 2.1. Energy Supply for Oocyte Maturation and Early Embryogenesis

Mitochondria are the principal source of ATP in the oocyte, generating energy through oxidative phosphorylation via the electron transport chain embedded in the inner mitochondrial membrane [3]. The energy demands placed on the oocyte are particularly intense during three sequential phases: meiotic maturation, fertilization, and the initial cleavage divisions of the preimplantation embryo [5].

During oocyte maturation, a dramatic surge in mitochondrial biogenesis occurs, substantially increasing mitochondrial copy number to meet the escalating energetic requirements of nuclear maturation, cortical granule exocytosis, and zona pellucida hardening [21]. Mature human oocytes contain an estimated 100,000 to 600,000 mitochondrial DNA (mtDNA) copies by far the highest of any human cell type reflecting the extraordinary energetic investment required to produce a fertilization-competent egg [21]. Studies have consistently demonstrated that both the quantity and the distribution of mitochondria within the oocyte are crucial determinants of developmental competence, with insufficient mitochondrial mass directly associated with fertilization failure and poor embryo development [4,6].

Following fertilization, new mitochondrial biogenesis is suppressed during the early cleavage divisions, and the existing maternal mitochondrial pool is simply partitioned among daughter blastomeres [22]. Each blastomere therefore inherits a progressively smaller mitochondrial quota, making the adequacy of the original oocyte mitochondrial reserve critically important for supporting embryo development through to the blastocyst stage [23]. A transient decrease in mtDNA copy number has been observed before blastulation in human embryos, suggesting that qualitative improvements in existing mitochondria are necessary to sustain the energy demands of this period [24].

Age-related decline in oocyte mitochondrial mass and function is well documented and represents a key biological mechanism underlying the steep drop in female fertility after the mid-thirties [11]. As oocytes age, the capacity for mitochondrial biogenesis decreases, ATP production falls, and the pool of functional mitochondria within the oocyte diminishes progressively [9]. This energetic deficit has direct consequences for every downstream reproductive process, from spindle assembly through to blastocyst formation and implantation [8].

### 2.2. Calcium Signalling and Meiotic Integrity

Beyond ATP production, mitochondria play an indispensable role in regulating intracellular calcium (Ca^2+^) homeostasis within the oocyte a function with profound consequences for meiotic progression and fertilization competence [25]. The mitochondrial calcium uniporter (MCU) facilitates the rapid uptake of Ca^2+^ into the mitochondrial matrix, where it stimulates key dehydrogenases of the tricarboxylic acid (TCA) cycle, thereby coupling calcium signalling directly to energy production during periods of high metabolic demand [26].

During meiotic maturation, mitochondria redistribute actively from a diffuse cytoplasmic pattern in germinal vesicle stage oocytes toward the developing meiotic spindle and cortical regions, establishing a localized ATP supply precisely where chromosomal movement and spindle dynamics require it most [27,28]. This strategic repositioning is critically dependent on intact mitochondrial dynamics the coordinated balance between fusion, mediated by mitofusin 1 (MFN1) and mitofusin 2 (MFN2), and fission, mediated by dynamin-related protein 1 (DRP1) [29]. Disruption of either process compromises mitochondrial distribution, impairs spindle assembly, and increases the risk of chromosome segregation errors leading to aneuploidy [30].

Specific deletion of MCU in mouse oocytes results in reduced mitochondrial Ca^2+^ concentrations, low ATP levels, abnormal spindle assembly, and disrupted meiotic progression directly demonstrating the mechanistic link between mitochondrial calcium handling and chromosomal integrity [26]. The clinical relevance of this relationship is substantial. Aneuploidy the leading cause of implantation failure, miscarriage, and birth defects in humans increases sharply with maternal age, and mitochondrial dysfunction is now recognized as a primary upstream driver of this phenomenon [31,32].

### 2.3. Redox Balance and Oocyte Longevity

A third dimension of mitochondrial function in the oocyte concerns the maintenance of redox balance the tightly regulated equilibrium between reactive oxygen species (ROS) production and antioxidant defense [13]. While physiological levels of ROS serve as important signalling molecules in oocyte maturation and fertilization, excessive ROS generation causes oxidative damage to lipids, proteins, and DNA, with mtDNA being particularly vulnerable due to its proximity to the electron transport chain and the limited repair mechanisms available in the mitochondrial compartment [33].

Immature oocytes in primordial follicles employ a remarkable and evolutionarily conserved strategy to minimize ROS accumulation during the prolonged period of dormancy: they suppress complex I of the mitochondrial electron transport chain, the primary site of superoxide generation, while maintaining sufficient ATP synthesis through alternative pathways [34]. Antioxidant defence through superoxide dismutase 1 (SOD1) further reinforces this low-ROS environment in dormant oocytes [35]. As oocytes age, this protective balance breaks down. Mitochondrial dysfunction generates increased ROS, which damages mtDNA, further impairing electron transport chain efficiency and producing yet more ROS a self-amplifying cycle of oxidative deterioration [36].

The consequence of sustained oxidative stress is not limited to acute cellular damage. Accumulating mtDNA mutations and deletions over time lead to impaired mitochondrial function across subsequent cell generations, with the potential for these defects to be transmitted through the maternal lineage and influence offspring health [37,38]. Taken together, the evidence reviewed in this section establishes that mitochondria are not simply energy providers in the oocyte but are central integrators of the metabolic, signalling, and genomic processes upon which successful reproduction depends. Any therapeutic strategy that meaningfully addresses oocyte quality must therefore engage directly with mitochondrial function (Figure 1).

## 3. Chemical Interventions Targeting Mitochondrial Function: Promise and Limitations

**Figure 2:**
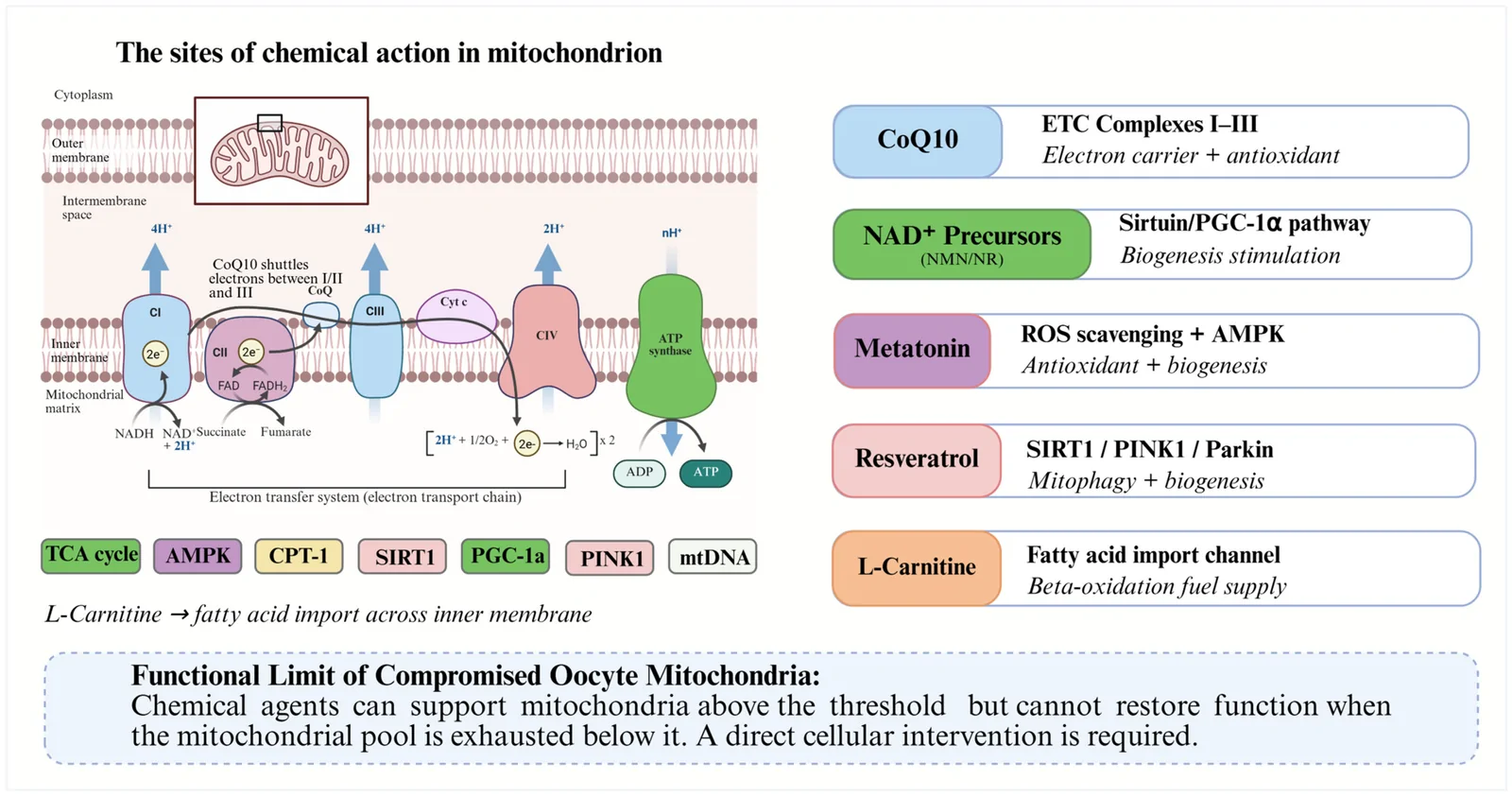
**Chemical Interventions: Mitochondrial Targets and Therapeutic Ceiling.** Schematic of the mitochondrial electron transport chain showing the site of action of each chemical intervention discussed in Section 3, with principal downstream effectors indicated below. The panel at right summarises the molecular target and functional effect of each agent. The boxed statement indicates the functional limit of compromised oocyte mitochondria. AMPK, AMP-activated protein kinase; ATP, adenosine triphosphate; CoQ10, coenzyme Q10; CPT-1, carnitine palmitoyltransferase 1; Cyt c, cytochrome c; ETC, electron transport chain; mtDNA, mitochondrial DNA; NAD^+^, nicotinamide adenine dinucleotide; NMN, nicotinamide mononucleotide; NR, nicotinamide riboside; PGC-1α, peroxisome proliferator-activated receptor gamma coactivator 1-alpha; PINK1, PTEN-induced kinase 1; ROS, reactive oxygen species; SIRT1, sirtuin 1; TCA, tricarboxylic acid.

Given the well-established link between mitochondrial dysfunction and poor oocyte quality, considerable research effort has been directed toward pharmacological and nutritional strategies that improve the mitochondrial microenvironment within reproductive cells [14]. These chemical interventions primarily antioxidants and metabolic cofactors represent the most clinically accessible tools currently available in ART practice (Figure 2), and several have demonstrated meaningful biological effects in both preclinical and clinical settings [39]. However, these agents share a common biological ceiling, discussed fully in Section 3.6.

### 3.1. Coenzyme Q10

Coenzyme Q10 (CoQ10) is a lipid-soluble quinone naturally present in the inner mitochondrial membrane, where it functions as an essential electron carrier between complexes I and II and complex III of the electron transport chain, while simultaneously serving as a potent endogenous antioxidant [40]. CoQ10 levels decline significantly with age in both ovarian tissue and peripheral cells, a pattern that parallels the age-related deterioration in oocyte mitochondrial function and reproductive potential [9]. Supplementation with CoQ10 has been shown to restore mitochondrial membrane potential, increase ATP production, and reduce oxidative damage to mtDNA in aging oocytes in murine models [41]. In human clinical studies, CoQ10 supplementation in women undergoing IVF has been associated with improvements in ovarian response, oocyte quality markers, fertilization rates, and embryo morphokinetics, though results across trials have not been uniformly consistent [42,43]. Despite these encouraging findings, the clinical utility of CoQ10 is constrained by its poor oral bioavailability and cannot compensate for an insufficient mitochondrial mass a distinction that becomes critical in aged oocytes where mitochondrial copy number itself is severely reduced.

### 3.2. Melatonin

Melatonin is a pineal hormone widely recognized for its chronobiological functions, but its role in reproductive biology has attracted increasing attention due to its potent antioxidant and anti-inflammatory properties [44]. Within the ovarian follicle, melatonin concentrations are substantially higher than in peripheral circulation, suggesting active local secretion and a dedicated role in protecting the oocyte from oxidative stress during follicular development [45,46]. Melatonin has been shown to improve oocyte maturation rates, reduce spindle abnormalities, and enhance blastocyst formation in both animal models and human IVF cycles [47,48]. Its capacity to activate AMPK and stimulate mitochondrial biogenesis through upregulation of nuclear respiratory factors and mitochondrial transcription factor A (TFAM) provides an additional mechanism beyond simple ROS scavenging [49]. However, as with CoQ10, the evidence base for melatonin in improving live birth rates in unselected ART populations remains insufficient, and the oocyte-protective effects of melatonin are necessarily limited to the mitochondria already present.

### 3.3. Resveratrol

Resveratrol is a naturally occurring polyphenol found principally in grapes and red wine, with well-characterized antioxidant and anti-inflammatory properties and an emerging role as an activator of sirtuin-dependent pathways that regulate mitochondrial biogenesis and quality control [50]. Through activation of SIRT1 and downstream induction of PGC-1α, resveratrol promotes mitochondrial biogenesis and stimulates mitophagy via upregulation of PTEN-induced kinase 1 (PINK1) and Parkin [51]. Preclinical studies have demonstrated that resveratrol supplementation improves oocyte quality in aged mice and reduces spindle abnormalities [52]. In women with PCOS, a randomized controlled trial reported improved mitochondrial biogenesis markers in granulosa cells and better ART outcomes with resveratrol supplementation [53]. The principal limitation remains its very low oral bioavailability [50], and as with other chemical interventions, resveratrol cannot address the fundamental deficit in mitochondrial mass that characterizes the aged or diseased oocyte.

### 3.4. L-Carnitine

L-carnitine is an endogenous amino acid derivative that plays an essential role in mitochondrial metabolism by facilitating the transport of long-chain fatty acids across the inner mitochondrial membrane for beta-oxidation [54]. L-carnitine supplementation has been shown to improve mitochondrial function in oocytes, enhance ATP production, and improve blastocyst formation rates in animal models and IVF patients [54,55]. Notably, clinical data demonstrated that oral L-carnitine administration prior to IVF improved embryo quality and clinical pregnancy rates, and a subsequent study confirmed that L-carnitine treatment reversed impaired mitochondrial function in human embryos as measured by oxygen consumption rate [56,57]. These findings are particularly relevant as they provide a coherent framework in which chemical and cellular mitochondrial interventions can be understood as complementary strategies within the same clinical program.

### 3.5. NAD^+^ Precursors

Nicotinamide adenine dinucleotide (NAD^+^) is an essential coenzyme in cellular energy metabolism and a critical cofactor for sirtuins the family of deacetylase enzymes that regulate mitochondrial biogenesis, DNA repair, and stress responses [58]. NAD^+^ levels decline significantly with age in ovarian tissue, contributing to age-related mitochondrial dysfunction and reproductive decline [6]. Supplementation with NAD^+^ precursors principally nicotinamide mononucleotide (NMN) and nicotinamide riboside (NR) has produced compelling results in preclinical reproductive models, improving oocyte quality, mitochondrial function, blastocyst formation rates, and live birth rates in aged animals [59]. More recently, NMN has been shown to enhance bovine oocyte developmental competence by improving mitochondrial function and reducing chromosome lagging during maturation [60] a finding that further substantiates the mechanistic basis for NAD^+^ supplementation. Early human data suggest potential benefits in IVF patients, though larger randomized trials are needed.

### 3.6. The Fundamental Limitation of Chemical Interventions and the Case for Cellular Therapy

The agents reviewed in this section CoQ10, melatonin, resveratrol, L-carnitine, and NAD^+^ precursors each target distinct but complementary aspects of mitochondrial function and together offer a rational multi-modal approach to supporting mitochondrial health in the oocyte. However, a critical biological boundary limits what chemical interventions can achieve. When mitochondrial dysfunction in the oocyte has progressed to the point where mtDNA copy number is critically reduced, mitochondrial membrane potential is severely compromised, or the proportion of functionally intact mitochondria falls below the threshold required to sustain the energy demands of maturation and embryogenesis, no pharmacological agent can substitute for the mitochondria themselves [6]. This is precisely the situation encountered in oocytes from women of advanced reproductive age, from patients with poor ovarian reserve, or following repeated ART failures the clinical scenarios where new therapeutic options are most urgently needed. It is this fundamental limitation that motivates the development of direct cellular mitochondrial interventions.

## 4. Cellular Mitochondrial Interventions: From Replacement to Transplantation

Building on the fundamental limitation of chemical interventions established in Section 3.6, attention now turns to cellular strategies that introduce healthy, functional mitochondria directly into the oocyte. Two fundamentally distinct approaches have been developed to this end: mitochondrial replacement therapy (MRT), which involves the use of donor-derived mitochondria through nuclear transfer techniques, and mitochondrial transplantation therapy (MTT), which introduces isolated mitochondria from a defined cell source into the recipient oocyte [15,61]. While both approaches address the core problem of mitochondrial insufficiency more directly than any chemical intervention can, they differ profoundly in their biological basis, safety profile, ethical implications, and clinical applicability [62].

### 4.1. Heterologous Mitochondrial Replacement Therapy: Scientific Achievement and Clinical Constraints

Mitochondrial replacement therapies represent a remarkable technical achievement in reproductive medicine. Techniques including maternal spindle transfer (MST), pronuclear transfer (PNT), and polar body transfer (PBT) each achieve the same fundamental objective: replacing the mitochondria of an affected oocyte or embryo with those from a healthy donor, while preserving the nuclear genetic identity of the intended parents [63]. These approaches were developed principally to prevent the maternal transmission of severe mitochondrial diseases caused by pathogenic mtDNA mutations [37].

The scientific rationale for MRT in this context is sound. MST has shown high rates of efficient mtDNA replacement [64], and a landmark pilot study reported six live births from 28 treatment cycles in patients with repeated IVF failure [65]. In this study, they have reported live births from MST in women with repeated IVF failure, suggesting that MRT may have reproductive applications beyond mitochondrial disease prevention though the ethical and regulatory constraints discussed below remain significant barriers to broad clinical adoption. PNT has similarly demonstrated blastocyst formation rates comparable to control embryos when performed within an optimal time window [66]. However, MRT carries biological, ethical, and regulatory constraints that severely limit its broader application [62,67].

**Figure 3:**
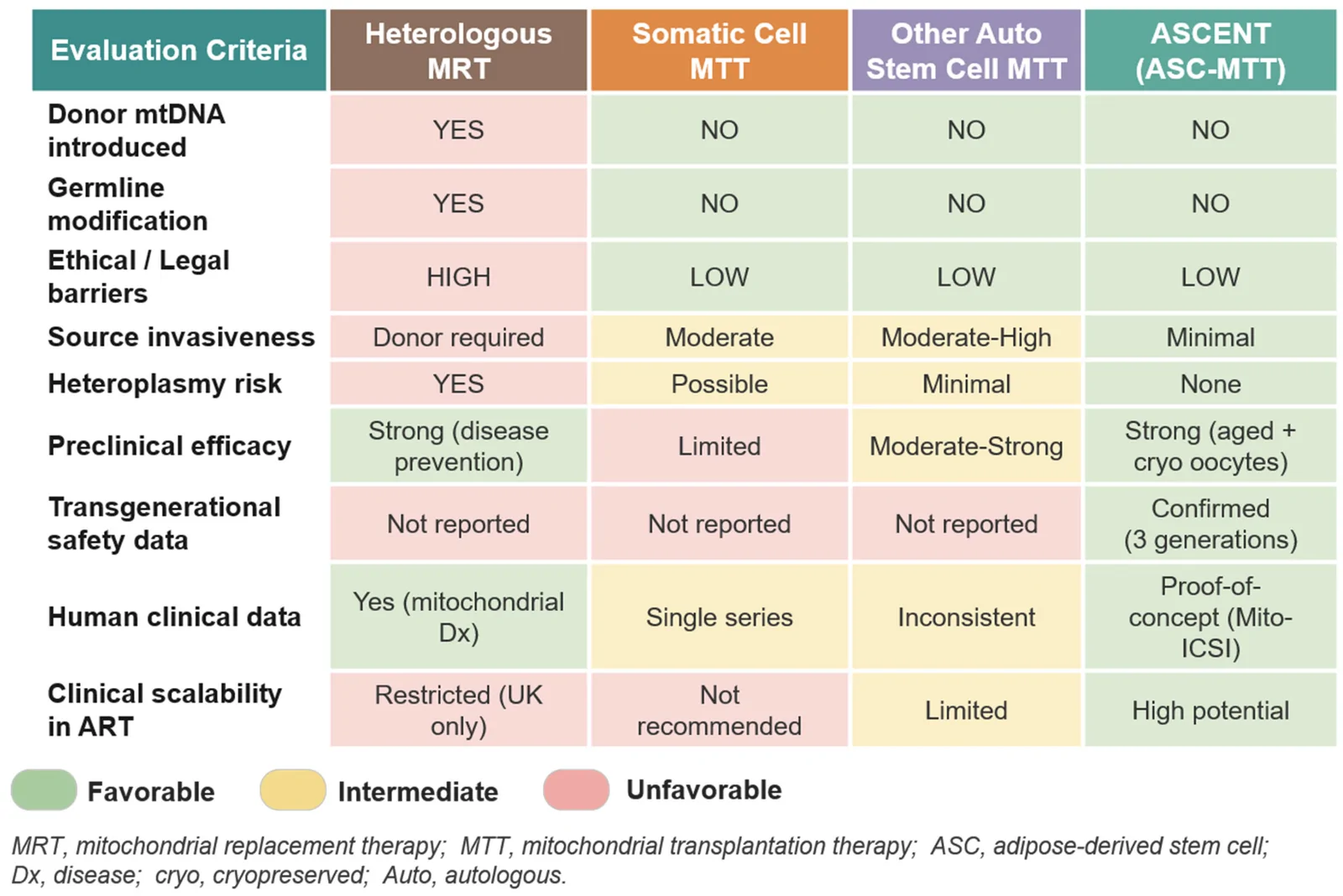
**Comparative Overview of Mitochondrial Intervention Strategies.** A structured comparison of four approaches; Heterologous MRT, Somatic Cell MTT, Other Autologous Stem Cell MTT, and ASCENT; across key parameters including donor mtDNA introduction, germline modification, ethical/regulatory barriers, invasiveness of cell source collection, strength of preclinical efficacy evidence, availability of human clinical data, and transgenerational safety characterization.

Biologically, MRT introduces mitochondria from a genetically distinct donor, creating a “three-parent” configuration [68]. The potential nuclear–mitochondrial DNA incompatibility in humans remains under investigation, but experimental evidence from animal models suggests that mismatched nuclear and mitochondrial genomes can impair metabolic efficiency and alter developmental outcomes [69,70]. Ethically and legally, MRT constitutes germline modification in the most literal sense the mitochondrial genome of the resulting child and all subsequent maternal-line descendants is permanently altered [71]. As of the time of writing, clinical MRT is legally permitted only in the United Kingdom, and exclusively for the purpose of preventing severe mitochondrial disease not for addressing age-related or idiopathic infertility [62]. These constraints make clear that while MRT represents a scientifically important advance for a specific indication, it is not a scalable solution for the broader challenge of mitochondrial dysfunction in ART (Figure 3).

### 4.2. Somatic Cell Mitochondrial Transfer: A Limited Interim Step

An intermediate approach transferring mitochondria isolated from the patient’s own somatic cells into her oocytes was explored as a means of preserving the autologous principle while bypassing the need for a donor [67]. The earliest clinical application reported reductions in apoptosis and fragmentation rates, improved fertilization rates, and increased live birth rates in a selected patient group [72]. However, somatic cell mitochondrial transfer carries a critical concern: somatic cells accumulate mtDNA mutations throughout life at rates substantially higher than germline cells, and transferring these mitochondria into oocytes raises the possibility of transmitting acquired mutations to offspring [73,74]. Furthermore, somatic cell mitochondria may not integrate effectively within the distinct metabolic environment of the oocyte [75]. These concerns have led to broad scientific consensus that somatic cell mitochondrial transfer is not an appropriate or sufficiently safe platform (Figure 3), for clinical ART application [76].

### 4.3. The Autologous Stem Cell Advantage: Why Source Matters

The use of stem cell-derived mitochondria for autologous transfer into oocytes addresses the principal limitations of both heterologous MRT and somatic cell transfer simultaneously [77]. Stem cells maintain mitochondria that are functionally young, characterized by high membrane potential, efficient oxidative phosphorylation, and a relatively low burden of accumulated mtDNA mutations [78]. From an ethical and regulatory standpoint, autologous stem cell MTT avoids entirely the concerns that attend heterologous MRT. Because the mitochondria originate from the patient’s own cells, no donor mtDNA is introduced, no heteroplasmy is created, and no germline modification in the heritable sense occurs [18]. The resulting child carries only the mitochondrial DNA of its biological mother precisely as in natural conception while benefiting from a supplemented mitochondrial pool within the oocyte [17].

Various stem cell sources have been investigated, including oogonial stem cells (OSCs), bone marrow-derived mesenchymal stem cells, umbilical cord-derived mesenchymal stem cells, endometrial stem cells, and induced pluripotent stem cells (iPSCs) [16,79,80]. Each has demonstrated some capacity to improve oocyte developmental outcomes in preclinical models. However, each also carries practical limitations that constrain clinical translation, summarised in Table 1 [81,82]. It should be noted that the comparative superiority of any single source in human clinical settings remains to be established empirically; the preclinical and clinical evidence base for each is compared in Table 1 [38,80]. Among all candidate sources evaluated to date, adipose-derived stem cells (ASCs) have emerged as uniquely well-suited for autologous mitochondrial transplantation in the ART context (Figure 3).

### 4.4. Why Adipose-Derived Stem Cells Stand Apart

Adipose tissue is the most accessible source of adult stem cells in the human body, obtainable in meaningful quantities through minimally invasive lipoaspiration under local anaesthesia [83]. Adipose tissue collection can be scheduled to coincide with oocyte retrieval, enabling same-day mitochondrial isolation and transfer without requiring a separate procedure cycle [16]. Beyond accessibility, ASCs exhibit significantly higher mitochondrial membrane potential and lower baseline ROS levels than other mesenchymal stem cell populations, reflecting a state of efficient, low-stress oxidative metabolism [16,84].

A particularly compelling finding that distinguishes ASC-derived mitochondria from other candidate sources is their morphological resemblance to oocyte mitochondria. Electron microscopic analysis has demonstrated that mitochondria isolated from murine ASCs display an oval morphology with limited cristae development a structural profile strikingly similar to that of mitochondria in mature oocytes [16,85]. This morphological compatibility suggests a degree of functional congruence that may facilitate integration and sustained activity following transplantation into the oocyte environment. The combination of practical accessibility, superior mitochondrial quality, morphological compatibility with oocyte mitochondria, and inherent immunological safety positions ASCs as the most clinically rational and scientifically substantiated source of mitochondria for autologous transplantation into human oocytes. The preclinical evidence generated using ASC-derived mitochondria, and the specific ASCENT approach that has emerged from this work, are examined in detail in the following section.

## 5. ASCENT: From Preclinical Evidence to Clinical Milestone

The preceding sections have established a coherent and evidence-driven argument: mitochondria are the central determinants of oocyte quality; chemical interventions are subject to the biological ceiling established in Section 3.6; heterologous replacement raises ethical and biological concerns that preclude broad clinical application; somatic cell transfer carries unacceptable mutation transmission risk; and among all autologous stem cell sources evaluated, adipose-derived stem cells possess the mitochondrial profile, practical accessibility, and morphological compatibility that make them uniquely suited for oocyte mitochondrial supplementation. A compelling candidate integrating these insights is ASCENT a strategy designed to be clinically translatable, ethically sound, and supported by a growing preclinical evidence base [16,18].

### 5.1. The Scientific Foundation of ASCENT

The development of ASCENT was preceded by carefully designed preclinical studies that established the biological basis for using ASC-derived mitochondria to improve oocyte developmental competence. Key foundational preclinical evidence demonstrated that transfer of autologous mitochondria from adipose tissue-derived stem cells into oocytes from aged mice significantly improved oocyte quality, enhanced embryo development, and increased live birth rates compared to untreated aged controls [84,85]. This work established proof-of-concept that ASC-derived mitochondria can functionally rescue oocytes compromised by age-related mitochondrial decline a prevalent and clinically significant form of mitochondrial insufficiency in ART practice. Building directly on this foundation, subsequent work by Kankanam Gamage and colleagues demonstrated that ASC mitochondrial transplantation significantly improved the developmental potential of cryopreserved oocytes a clinically important application given the widespread use of oocyte vitrification in contemporary ART [16,85]. Critically, this study provided detailed mechanistic insight: transplantation of ASC-derived mitochondria was shown to enhance the ATP production capacity of embryos without concomitant increases in ROS levels [16,85]. This pattern improved energy efficiency without increased oxidative stress is precisely the opposite of what occurs in aged or cryoinjured oocytes, and it suggests that the transplanted ASC mitochondria are not simply adding to total mitochondrial mass but are actively improving the quality and efficiency of mitochondrial energy metabolism within the oocyte.

### 5.2. Transgenerational Safety: The Critical Evidence

Among all the evidence supporting ASCENT, the most scientifically distinctive and clinically significant finding concerns transgenerational safety. A dedicated transgenerational safety study evaluated the effects of autologous ASC mitochondrial supplementation across three consecutive generations of mice the most comprehensive safety assessment yet performed for any form of mitochondrial transplantation in a reproductive context. This study, which formally introduced the ASCENT designation, demonstrated that offspring born following ASC mitochondrial transfer showed no significant adverse effects on health, growth, behavior, or reproductive performance compared to wild-type controls findings that held consistently across the first, second, and third filial generations [16,18].

Among published autologous MTT approaches, no comparable three-generation safety study has been reported to date though it should be noted that other approaches remain at earlier stages of development, and the absence of such data should not be equated with demonstrated risk [86] Analysis of the mtDNA origin in offspring born following ASCENT confirmed that the mitochondrial DNA present in these animals was derived primarily from the maternal oocyte rather than from the transplanted ASC mitochondria. This finding is biologically reassuring on multiple levels: it confirms that ASCENT does not create a stable heteroplasmic state in offspring, and that the intervention achieves its beneficial effects without permanently altering the mitochondrial genetic identity of the resulting individual [16,18,84].

These findings define the current evidence horizon rather than a complete long-term safety profile, and three limitations warrant explicit acknowledgement. First, because the approach is autologous, it does not generate heteroplasmy in the donor–recipient mis-match sense that has constrained heterologous MRT: the transplanted mitochondria and the recipient oocyte share a single nuclear background, so classical mitonuclear incompatibility does not arise. This is a genuine biological distinction rather than an absence of data. Second, the observation that offspring mtDNA derives primarily from the maternal oocyte does not exclude persistence of a minor ASC-derived population, and the copy-number dynamics of transplanted mitochondria across embryonic, fetal and post-natal development have not been quantitatively characterised; whether such a population is progressively diluted, stably maintained or selectively amplified in specific tissues re-mains unknown. Third, adipose-derived mitochondria originate from a somatic lineage that has not passed through the germline bottleneck and may therefore carry age-acquired mtDNA variants absent from the oocyte’s own population, reinforcing the need for the pre-use quality screening outlined in Section 6.1. The long-term consequences of introducing such variants, if any, cannot be resolved by a three-generation murine dataset spanning approximately two years, a horizon short relative to the multi-decade timescale implicit in a human safety claim. These limitations are not grounds for withholding clin-ical evaluation, but they define a specific and tractable research agenda: prospective mtDNA quantification in offspring tissues, and structured longitudinal follow-up ex-tending into adulthood (Section 6.4).

### 5.3. Clinical Translation: The ASCENT Workflow

A significant practical advantage of ASCENT lies in its clinical workflow, which has been designed for integration into standard IVF/ICSI practice without requiring additional patient procedures or cycle delays. The ASCENT procedure unfolds in three coordinated steps aligned with the existing ART cycle. First, adipose tissue is collected from the patient through minimally invasive lipoaspiration under local anaesthesia. Second, mitochondria are isolated from the processed ASCs using a standardized differential centrifugation protocol, with quality assessment including membrane potential measurement performed prior to use. Third, on the day of ICSI, the characterized ASC-derived mitochondria are co-injected with the selected spermatozoon into the mature metaphase II oocyte a modification of the standard ICSI procedure that adds minimal time and complexity [16,18].

This workflow preserves the autonomy and genetic integrity of the patient throughout. The primary candidates for ASCENT in clinical practice would include women of advanced reproductive age with poor oocyte quality despite adequate ovarian response, patients with repeated IVF failure not explained by other factors, and women with poor embryo development in the context of good fertilization rates [87]. These are precisely the patient groups for whom existing interventions have proven insufficient, and for whom no effective evidence-based alternative currently exists.

### 5.4. Clinical Evidence: Early Human Data

While the primary evidence base for ASCENT remains preclinical, early clinical data from the research group that developed the approach provide an important proof-of-concept signal in humans. Morimoto and colleagues reported the outcomes of autologous OSC-derived mitochondrial transfer into oocytes in patients with recurrent pregnancy failure, demonstrating improvements in embryo quality and live birth rates, with follow-up analysis of offspring confirming no significant developmental defects and primary maternal origin of the babies’ mitochondrial DNA [17,88,89,90]. While this clinical series used OSC-derived rather than ASC-derived mitochondria, it was performed by the same group using the same Mito-ICSI delivery methodology that underpins ASCENT. It therefore provides relevant evidence of technical feasibility and acceptable safety for autologous Mito-ICSI as a delivery platform but it does not constitute direct clinical evidence for ASCENT specifically (which uses ASC-derived mitochondria), and a dedicated first-in-human ASCENT study remains needed [17,88,89,90]. The convergence of the preclinical ASCENT evidence with this clinical proof-of-concept creates a compelling basis for proceeding to a formal first-in-human clinical evaluation of ASCENT.

### 5.5. ASCENT in the Landscape of Autologous MTT: A Comparative Perspective

To appreciate the specific contribution of ASCENT, it is useful to position it against other autologous stem cell MTT approaches, as summarized in Table 1. OSC-derived MTT has been the only other autologous stem cell approach to reach clinical application, with published series reporting generally positive effects on embryo quality [17,88,89,90]. However, OSC-based approaches carry significant limitations: the definitive biological existence of OSCs in adult human ovaries remains scientifically contested [81], the isolation procedure requires ovarian cortex biopsy, and a randomized controlled trial found no significant improvement following OSC-derived mitochondrial transplantation [89]. Endometrial stem cell MTT has shown promise in aged murine models [79] but requires an endometrial biopsy in a separate cycle. iPSC-derived MTT has shown impressive results in aged murine models [80] but biosafety concerns and regulatory complexity create substantial barriers to near-term translation.

Against this landscape, it was performed by the same group using the same Mito-ICSI delivery methodology that underpins ASCENT. It therefore provides relevant evidence of technical feasibility and acceptable safety for autologous Mito-ICSI as a delivery platform but it does not constitute direct clinical evidence for ASCENT specifically (which uses ASC-derived mitochondria), and a dedicated first-in-human ASCENT study remains needed: [16,17].

### 5.6. Outstanding Questions and the Path to Clinical Validation

Designating ASCENT as a milestone does not imply that all questions have been resolved. Several questions require systematic investigation in the transition from preclinical models to human clinical application. The optimal number of mitochondria to transfer per oocyte has not yet been established for the human clinical context, and determining the dose-response relationship will be an important early objective [77,91]. The standardization of mitochondrial isolation protocols requires formalization to ensure consistency across different laboratory settings [75]. The criteria for patient selection need to be defined through appropriately designed clinical studies [87].

The mechanism by which a relatively small number of transplanted mitochondria produces disproportionately large improvements in oocyte and embryo function. remains incompletely resolved; the leading hypotheses and their respective evidentiary basis are examined critically in Section 6.3. Despite these open questions, the evidence base assembled is sufficient to justify the next step: a rigorously designed, prospective, controlled first-in-human clinical trial of ASCENT in an appropriately selected patient population.

## 6. Challenges and Future Directions: Translating ASCENT into Clinical Practice

The scientific case for ASCENT is compelling, and the convergence of preclinical efficacy data, mechanistic insight, transgenerational safety confirmation, and human proof-of-concept through Mito-ICSI creates an unusually strong foundation for clinical translation. However, the responsible advancement of any novel intervention in reproductive medicine a field where the consequences of unanticipated harm extend not only to patients but to the children they conceive and potentially to subsequent generations demands that the path from preclinical promise to clinical practice be navigated with scientific rigor, methodological transparency, and ethical deliberation [18,84].

### 6.1. Standardization of Mitochondrial Isolation and Quality Assessment

The reproducibility of any cell-based therapeutic intervention depends fundamentally on the consistency of the biological material being administered. Currently, no universally accepted standard protocol exists for the isolation of mitochondria from ASCs intended for clinical use, and methodological variability across published preclinical studies limits the confidence with which any single protocol can be adopted for clinical application [16,85]. Differential centrifugation remains the most widely used mitochondrial isolation method due to its technical simplicity, but the mechanical shear forces involved can compromise outer mitochondrial membrane integrity and reduce functional viability of the isolated preparation [92]. Establishing which isolation methodology best preserves the functional characteristics of ASC-derived mitochondria intended for oocyte supplementation is a necessary prerequisite for clinical standardization. A comparative evaluation of the principal isolation methodologies is presented in Table 2.

Three isolation approaches are relevant to clinical translation, and they differ in ways that bear directly on product quality (Table 2). Differential centrifugation is the most widely used method and requires no specialised reagents, but it separates by sedimentation rate alone and therefore co-isolates lysosomes, peroxisomes, Golgi membranes and sarcoplasmic reticulum along with mitochondria; in direct comparison, only 59% of the resulting fraction was positive for translocase of outer mitochondrial membrane 22 (TOM22) by flow cytometry [93]. Density gradient centrifugation separates by buoyant density and yields substantially higher purity while preserving respiratory activity [94]. Gradient media are not, however, interchangeable for clinical purposes: iodixanol, developed as an intravascular radiographic contrast medium, is non-ionic, metabolically inert and manufactured under current good manufacturing practice conditions [95], and is therefore the more appropriate choice where a clinical-grade preparation is intended. An-ti-TOM22 immunomagnetic separation achieves both the highest reported yield and purity comparable to ultracentrifugation, with markedly shorter processing time [93,96], and is therefore attractive where starting material is limited. Closed-system immuno-magnetic separation has established manufacturing precedent in hematopoietic graft processing and chimeric antigen receptor T-cell production; no anti-TOM22 reagent, however, is currently released to good manufacturing practice standards for a clinical mitochondrial product, and no validated closed-system protocol has been reported for any of these three methods. Microfluidic separation has been described for mitochondrial isolation at analytical scale, but we could identify no published data on yield, purity or functional preservation at the quantities required for clinical preparation, and it is therefore not included in the comparison below.

**Table 2:** Comparison of mitochondrial isolation methodologies for clinical translation. Reported for Percoll-based gradients in skeletal muscle; comparable data for iodixanol-based mitochondrial gradients were not identified. ^a^ Absence of a reported protocol; no citation available. ^b^ Withdrawn following endotoxin contamination concerns. DC, differential centrifugation; ER, endoplasmic reticulum; FACS, fluorescence-activated cell sorting; GMP, good manufacturing practice; OCR, oxygen consumption rate; RCR, respiratory control ratio; TOM22, translocase of outer mitochondrial membrane 22; UC, ultracentrifugation.

Parameter	Differential Centrifugation	Density Gradient	Anti-TOM22 Immunomagnetic
**Yield**	Baseline (reference) [93]	Comparable to DC [94]	~2 × DC; ~4 × UC [93]
**Purity (TOM22^+^, FACS)**	59% [93]	High; depleted of ER, lysosomal and Golgi markers [94]	89% [93]
**Functional preservation**	Variable; operator-dependent [92]	RCR 3.9–7.1 [94]	OCR confirmed by Clark electrode and flux analysis [96]
**Processing time**	Moderate; multiple cycles [93]	Extended; gradient layering [94]	1–2 h from homogenization [93]
**GMP scalability**	No validated closed-system protocol reported ^a^	Iodixanol manufactured to cGMP [95] Percoll withdrawn from clinical ART use, 1996 ^b^ [97]	Platform precedent in cell therapy; no GMP-released anti-TOM22 reagent ^b^

Equally important is the development and validation of a robust quality control framework for the mitochondrial preparation prior to clinical use. Mitochondrial mem-brane potential, as assessed by fluorescent dye exclusion, is the most commonly reported quality metric and correlates with respiratory chain function and ATP-generating capacity [98]. Whether membrane potential alone is sufficient as a release criterion, or whether additional parameters including oxygen consumption rate, mtDNA copy number, and ROS generation should be incorporated into a composite quality score, requires prospective evaluation [86]. A proposed multi-parametric release-criteria panel, extending beyond membrane potential alone, is presented in Table 3.

Membrane potential is the parameter most frequently reported, but it is an incomplete indicator when considered alone. Potentiometric dyes report the transmembrane electro-chemical gradient at the moment of measurement, and a reduced signal may reflect transient depolarization sustained during isolation rather than irreversible loss of respiratory competence; conversely, retained potential does not establish that coupled oxidative phosphorylation is intact. Interpreted alongside a direct measure of respiratory capacity, the parameter becomes considerably more informative. Preparation intended for clinical use requires assessment across several complementary domains: bioenergetic competence, quantitative dose, oxidative status, microbiological safety and freedom from non-mitochondrial material. A panel addressing each is proposed in Table 3. It should be stated plainly that these parameters have not been qualified as release criteria against clinical outcome data for mitochondrial preparations used in assisted reproduction, and that no consensus specification exists for this product class. The assays are individually well established, but the acceptance thresholds are not. Where a defined value could be drawn from an adjacent clinical cellular product its origin is indicated; where it could not, criteria are expressed relative to a validated reference preparation and require prospective establishment. Development and formal qualification of such assays represent a necessary step for the field, and the panel below is offered as a framework toward that end rather than as an implemented standard (Table 3).

The immunological risk profile of isolated mitochondria warrants analysis specific to the oocyte, since the pathways involved are characterised almost entirely in somatic and immune cell contexts. Mitochondria retain features of their bacterial ancestry and, if their membranes are compromised during isolation or microinjection, can act as damage-associated molecular patterns (DAMPs) through three principal sensing routes [99]. Unmethylated CpG motifs in released mtDNA engage endosomal Toll-like receptor 9 (TLR9), signalling through myeloid differentiation primary response 88 (MyD88) to activate nuclear factor kappa B (NF-κB) and interferon regulatory factors [99]. Cytosolic mtDNA and mitochondrial reactive oxygen species activate the NOD-like receptor protein 3 (NLRP3) inflammasome, driving caspase-1-dependent maturation of interleukin-1β and interleukin-18 [101]. Cytosolic mtDNA also engages cyclic GMP-AMP synthase (cGAS), activating the stimulator of interferon genes (STING) axis and a type I interferon response [102].

Applying these pathways to the oocyte requires care, and the following should be read as a reasoned risk model rather than a demonstrated finding: to our knowledge, no study has characterised DAMP signalling following isolated mitochondrial microinjection in oocytes or early embryos. Two features of this microenvironment are nonetheless relevant. The metaphase II oocyte is transcriptionally quiescent, so a canonical interferon response is unlikely to originate from the oocyte itself; the surrounding cumulus and granulosa cells, which express Toll-like receptors and inflammasome components, represent the more plausible responding compartment, and inflammasome-driven pyroptosis in that compartment could compromise the transzonal projections on which cytoplasmic maturation depends. Following fertilisation, embryonic genome activation restores transcriptional competence, so interferon-mediated responses become theoretically possible at cleavage stages. Two considerations temper this risk. Reported injection volumes are 1–10 pL (Table 4), orders of magnitude below the mitochondrial loads used in somatic transplantation studies in which DAMP responses have been observed. More fundamentally, DAMP exposure is a function of preparation quality rather than of mitochondrial transfer per se, since intact organelles sequester mtDNA and N-formyl peptides within their membranes. The membrane-integrity, purity and endotoxin criteria specified in Table 3 therefore constitute the principal practical mitigation, and this immunological rationale is a substantive argument for adopting them as formal release criteria rather than research-grade characterization.

### 6.2. Determining Optimal Mitochondrial Dose

The question of how many mitochondria to transfer per oocyte is among the most practically important unresolved issues in ASCENT translation [75]. Preclinical studies have employed a range of mitochondrial quantities, making cross-study dose comparison difficult, and the dose–response relationship between the number of transplanted mitochondria and functional oocyte outcomes has not been systematically characterized. Underdosing would predictably result in no detectable clinical benefit, potentially generating false-negative signals in early clinical trials [67,75]. Overdosing risks disrupting the oocyte’s existing mitochondrial network dynamics [29]. A further dimension concerns variation in baseline mitochondrial status across individual patient oocytes. The development of validated non-invasive or minimally invasive biomarkers of oocyte mitochondrial status would enable individualized dosing strategies aligned with each patient’s specific mitochondrial deficit [105,106]. The heterogeneous dosing reported across existing preclinical and clinical studies, compiled from the primary literature, is presented in Table 4.

Reported doses are summarised in Table 4. Injected volumes fall within a relatively narrow range of 1 to 10 pL across studies, but volume alone does not establish comparable exposure, since the delivered number of organelles also depends on the concentration of the preparation. Preparation concentration is reported inconsistently across this literature, variously as source cell number per unit volume, as total mitochondrial protein concentration, or as the proportion of oocyte volume occupied, and conversion between these conventions requires isolation yield figures that are not consistently reported. Only two studies quantified what was actually delivered, and these used different units again: ap-proximately 788 mtDNA copies per oocyte in one [104] and approximately 500 mitochondria per oocyte in the other [89]. Cross-study dose comparison is therefore not reliably possible from the published data, irrespective of how carefully each study was individually conducted. We propose that mitochondrial DNA copy number per injected volume be adopted as a reporting standard, this being an absolute measure, quantifiable at the point of delivery, and already included in the release panel proposed in Table 3. This is not a novel proposal: Cagnone et al. quantified their preparation in exactly this way, by injecting an identical volume into a reaction tube and measuring copy number by re-al-time PCR [104]. The variability of that measurement, 787.5 ± 409.3 copies, also illustrates why batch-level quality control matters. Adoption of a common unit would permit dose–response characterization across studies and centers, which is a prerequisite for rational dose selection in clinical evaluation.

### 6.3. Understanding the Mechanism of Action

One of the most scientifically intriguing unresolved questions about ASCENT concerns the mechanism by which a relatively modest supplementation of the oocyte’s mitochondrial mass produces the magnitude of functional improvement observed in preclinical models [16,18]. Three non-mutually exclusive hypothesis, each resting on a different evidentiary basis, have been proposed. The first holds that transplanted mitochondria act as a retrograde-signaling seed population: by locally shifting the AMP/ATP and NAD+/NADH ratios, they activate AMPK and SIRT1, which converge on PGC-1α to drive NRF1/2-TFAM-mediated biogenesis of the oocyte’s own endogenous mitochondrial pool [58], expanding functional mitochondrial mass well beyond the injected organelles themselves; this mechanism rests on well-established general mitochondrial biology but has not been directly demonstrated in the oocyte context. The second holds that mitochondria with intact fusion machinery merge, via MFN1, MFN2, and OPA1, with dysfunctional resident mitochondria, enabling complementation of mtDNA defects through mitochondrial content mixing [107]; this mechanism is supported by the broader mitochondrial dynamics literature but likewise remains inferential in this specific application. The third holds that transplanted mitochondria shift the oocyte’s mitochondrial network dynamics more broadly, moving the balance from the fragmented, fission-dominant state characteristic of aged oocytes toward a more fused, interconnected architecture [6,29]. At present, no ASCENT-specific data distinguish among these three mechanisms, and their relative contribution should be regarded as an open, high-priority research question rather than a resolved feature of the platform. Elucidating which mechanism predominates likely through oocyte-specific tracking of donor mitochondria (for example, via mtDNA haplotype discrimination) alongside biogenesis- and fusion-marker assays, will enable rational optimization of the approach and may open opportunities for pharmacological co-interventions that amplify the therapeutic effect [59,60].

### 6.4. Clinical Trial Design and Patient Selection

The translation of ASCENT into clinical practice will require a carefully designed prospective clinical trial. Patient selection criteria must be defined with sufficient precision to identify the population most likely to benefit from ASCENT while excluding patients whose poor ART outcomes are attributable to non-mitochondrial causes [87,108]. The most scientifically justified primary target population would include women of advanced reproductive age generally defined as 38 years and older with documented poor oocyte or embryo quality despite adequate ovarian response, or women with repeated unexplained IVF failure. Secondary populations of interest would include women with PCOS, endometriosis, or obesity in whom mitochondrial dysfunction has been specifically documented [13,105].

The primary endpoint of the initial clinical trial should provide a robust and biologically meaningful signal within a feasible study timeline. Blastocyst formation rate per oocyte retrieved represents a scientifically appropriate primary endpoint for a Phase I/II safety and efficacy study [24]. Live birth rate per initiated cycle remains the gold standard outcome and should be incorporated as a key secondary endpoint in subsequent Phase III evaluation [108]. A randomized sibling oocyte design offers several methodological advantages by controlling for inter-patient variability within each cycle and providing each patient with a contemporaneous internal control. Long-term follow-up of children born following ASCENT should be incorporated as a mandatory component of the protocol [18,86].

These design considerations operate under constraints that warrant explicit acknowledgement. Because no human efficacy data exist for adipose-derived mitochondrial supplementation, the appropriate first step is a pilot-level study powered to establish safety, procedural feasibility and preliminary biological signal, with expansion to a larger controlled trial contingent on those findings rather than assumed at the outset. The eligible population is inherently limited: patients must present a mitochondrial rather than a non-mitochondrial cause of poor outcome, and must be willing to undergo an additional operative procedure alongside a high-complexity cycle. Recruitment at a single centre is therefore likely to be slow and statistical power correspondingly modest, which argues for multicentre collaboration from the outset and for interpreting early findings as hypothesis-generating rather than confirmatory. Confounding presents a further difficulty: out-comes in this population vary with maternal age band, ovarian reserve, body mass index and underlying aetiology, and stratification across these variables is necessary if apparent treatment effects are not to reflect cohort composition. In a small cohort, stratification and statistical power are in direct tension, and this trade-off should be addressed at the design stage rather than in post hoc analysis. Patient selection is not merely a matter of trial efficiency: in porcine oocytes, mitochondrial supplementation improved blastocyst development in oocytes selected for mitochondrial DNA deficiency but conferred no benefit in oocytes of normal copy number [104]. If the same holds in humans, a cohort not selected for mitochondrial status could dilute a real effect below the threshold of detection. The sib-ling-oocyte design, while effective in controlling inter-patient variability, carries specific costs: it divides an already limited oocyte cohort between arms, reduces the number of embryos available for transfer decisions in each, and does not permit evaluation of patient-level endpoints such as live birth rate per initiated cycle, which require parallel-group randomization. Finally, long-term follow-up of offspring is subject to attrition, relocation and the requirement to obtain consent afresh at the age of majority. Follow-up commitments should therefore be structured as registry-based programs with institutional rather than single-sponsor continuity, and the realistic limits of such follow-up should be communicated to participants during consent rather than presented as an un-qualified assurance.

### 6.5. Regulatory Pathway and Ethical Framework

The regulatory classification of ASCENT will vary across jurisdictions and will significantly influence the pathway to clinical implementation. In many regulatory frameworks, a procedure involving the isolation of autologous cellular material and its reinstatement into the same patient without ex vivo cell culture or genetic modification may be classified as a minimal manipulation procedure, attracting a relatively streamlined regulatory pathway [62]. However, because ASCENT involves the introduction of isolated organelles into an oocyte that may subsequently be fertilized and give rise to a new individual, regulatory agencies may apply heightened scrutiny to the safety data required [15,16].

From an ethical standpoint, the autologous approach avoids several of the specific concerns that attend heterologous MRT, though it does not resolve the broader questions considered below. Because no donor genetic material is introduced and no heritable germline modification in the conventional sense occurs, the principal ethical concerns that have generated controversy and regulatory restriction around MRT do not apply with comparable force to ASCENT [71,109]. Nevertheless, comprehensive informed consent processes must clearly communicate the current state of evidence, the remaining uncertainties, and the planned monitoring provisions to prospective patients [110].

Frameworks governing mitochondrial manipulation in assisted reproduction differ substantially between jurisdictions and were, in most cases, drafted with heterologous mitochondrial replacement therapy (MRT) rather than autologous approaches in view. In the United Kingdom, the Human Fertilization and Embryology Authority operates a statutory licensing framework and has permitted MRT since 2015, restricted to prevention of transmissible mitochondrial disease under license at a designated centre [111,112]. Any manipulation of human oocytes or embryos requires an Authority license irrespective of whether the procedure meets the statutory definition of MRT.

In the United States, oversight has been constrained since December 2015 by an annually renewed appropriations provision prohibiting the Food and Drug Administration from reviewing any investigational application involving a human embryo intentionally created or modified to include a heritable genetic modification [112,113]. The provision was enacted in response to donor-based MRT, and its application to autologous approaches introducing no donor mitochondrial DNA has not been tested. Legal commentators have separately questioned whether MRT itself satisfies the heritable genetic modification threshold as drafted.

In China, the 2003 Ministry of Health Technical Standards and Ethical Principles for Human Assisted Reproduction Technology prohibit cytoplasm transfer and germinal vesicle transfer in infertility treatment, a provision directed at donor-derived material [111,114]. Oversight of germline modification has since broadened, and the 2024 Ministry of Science and Technology ethical guidelines impose a binding prohibition on heritable genome editing for reproductive purposes [111]. Neither instrument addresses autologous approaches that introduce no donor mitochondrial DNA.

In Japan, assisted reproductive technology is expressly excluded from the scope of the Act on the Safety of Regenerative Medicine, except where embryonic stem cells derived from human sperm or unfertilized oocytes are used [115]. This exclusion does not place assisted reproduction outside regulatory oversight. Practice is governed by binding Views issued by the Japan Society of Obstetrics and Gynecology, a self-regulatory system with documented enforcement precedent including revocation of society membership for guideline violation, reinforced by a mandatory national facility registry in continuous operation since 1986 and, since April 2022, by conditions attaching to public health insurance coverage [116]. No View currently addresses autologous mitochondrial supplementation specifically.

In South Korea, the Bioethics and Safety Act, effective from 2005 and most recently revised in 2020, governs human stem cell research and in vitro fertilization [117]. Its provisions are oriented toward embryo research and somatic cell nuclear transfer, and its application to autologous adult stem cell-derived products used in assisted reproduction has not been tested.

A consistent pattern emerges across these jurisdictions: statutory frameworks address donor-derived mitochondrial material and germline modification in detail, while autologous approaches introducing no donor mitochondrial DNA remain largely unaddressed at the level of specific guidance. This is not equivalent to permission. Where a technology falls outside the express scope of a statute, it ordinarily remains subject to general clinical research governance, institutional review and, in several of these jurisdictions, professional-body oversight. The absence of technology-specific rules should be read as regulatory immaturity rather than as an established route to clinical use, and clinical development in any of these settings warrants prior consultation with the relevant national authority or professional body.

The ethical position of autologous mitochondrial transfer nonetheless raises questions that a favorable comparison with MRT does not resolve, and these warrant direct consideration. The first concerns overmedicalization. Mitochondrial supplementation is not indicated for all patients with poor reproductive outcomes: in porcine oocytes, benefit was confined to those selected for mitochondrial DNA deficiency, with none observed in oocytes of normal copy number [104]. Applied without selection for mitochondrial status, the procedure would expose patients to an additional operative step and cost for no expected gain, which makes appropriate patient selection an ethical requirement rather than solely a question of trial design.

The second concerns commercial pressure. Novel autologous cellular technologies can be marketed to vulnerable patients in advance of robust efficacy evidence, and the field offers a direct precedent: a randomized study of oogonial stem cell-derived transfer found no improvement in euploidy rate (9.8% versus 11.9% per metaphase II oocyte, *p* = 0.541) and a significantly lower blastocyst formation rate in the treated arm (23.3% versus 41.1%) [89]. Although that protocol used a different cell source, the difference in source is not itself a reason to expect a different outcome, since no comparative human data exist. No human efficacy data yet exist for adipose-derived mitochondrial supplementation, and clinical offering in advance of such data would not be justified.

The third concerns proportionality. The approach adds an operative step and labor-atory processing to an already demanding treatment cycle, and where poor oocyte quality reflects chromatin or spindle-level defects rather than bioenergetic deficit, supplementa-tion would not be expected to confer benefit at any dose. The fourth concerns obligations to offspring. Follow-up registries spanning decades carry real cost, cannot depend on the continuity of a single sponsor or clinic, and raise a consent question that parental authorisation cannot settle, since the resulting individual must be free to decline participation on reaching majority.

### 6.6. Toward International Collaborative Clinical Development

Given the inherent challenges of recruiting sufficient patient numbers within a single center, international collaborative trial design represents both a practical necessity and a scientific opportunity for the initial clinical evaluation of ASCENT [17,108]. A multi-center trial involving experienced ART centers with documented capacity to perform complex micromanipulation procedures, access to adipose tissue collection facilities, and established protocols for mitochondrial quality assessment would maximize the pace of patient accrual and enhance the generalizability of findings [17]. Multi-center collaboration would also enable cross-center standardization of the ASCENT protocol to be evaluated and optimized in parallel with clinical efficacy assessment. The establishment of an international registry for ASCENT cases capturing procedural details, mitochondrial preparation characteristics, oocyte and embryo outcomes, and long-term follow-up data on children born through the procedure would complement formal trial data with real-world evidence and provide an ongoing safety surveillance mechanism as clinical experience accumulates [86,118]. This registry model has been successfully applied in other areas of reproductive medicine involving novel interventions, and its adoption for ASCENT would reflect best practice in the responsible introduction of innovative ART procedures. The scientific and clinical infrastructure required to support this collaborative development represents a significant organizational investment. It is, however, precisely the investment that the quality of the ASCENT evidence base and the magnitude of the unmet clinical need collectively justify. 

### 6.7. Beyond ART: Broader Regenerative Medicine Applications of ASC-Derived Mitochondria

**Figure 4:**
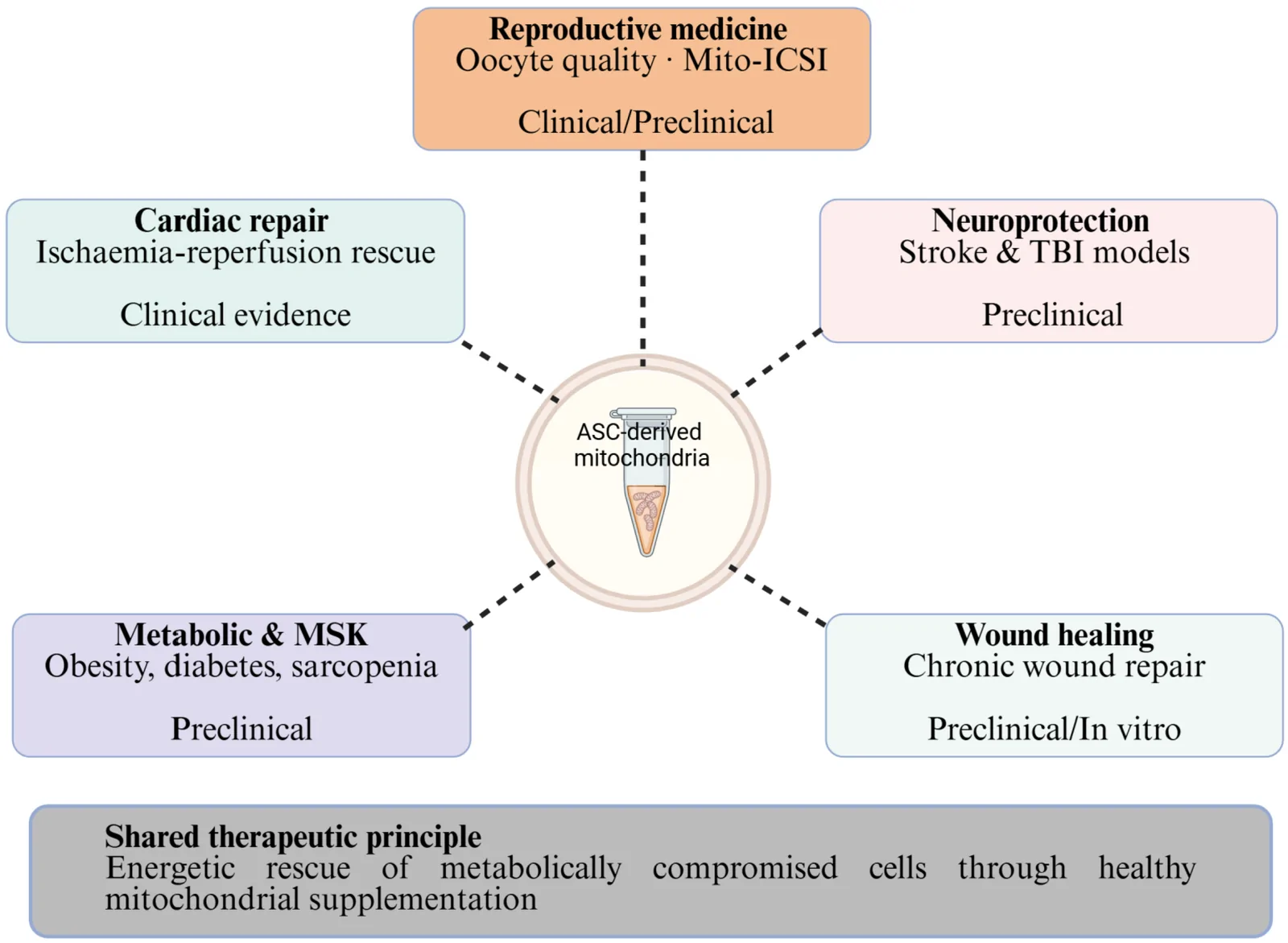
**ASC-Derived Mitochondria in Broader Regenerative Medicine.** A schematic illustrating the four major non-reproductive application contexts for ASC-derived mitochondrial transplantation: cardiac (ischaemia–reperfusion rescue), neurological (stroke/TBI neuroprotection), musculoskeletal (satellite cell support), and wound healing (keratinocyte energetic rescue). Arrows indicate the shared mechanistic principle energetic rescue of metabolically compromised cells and highlight that the ASCENT platform builds on this wider regenerative medicine evidence base.

The therapeutic potential of mitochondrial transplantation extends considerably beyond the oocyte and reproductive medicine. A growing body of evidence across multiple organ systems demonstrates that the introduction of healthy mitochondria into cells experiencing energetic failure can confer meaningful protective or restorative effects a principle that may broadly underpin ASC-derived mitochondrial therapy as a regenerative platform [119,120], and the ASCENT platform builds on this wider regenerative medicine evidence base (Figure 4).

#### 6.7.1. Cardiac Applications

The most clinically advanced applications of mitochondrial transplantation outside of reproductive medicine are in cardiac surgery. Seminal work by Masuzawa and colleagues demonstrated that transplantation of autologous mitochondria derived from skeletal muscle into ischemic myocardium significantly improved contractile function in a rabbit model of ischemia–reperfusion injury [121,122]. This approach was subsequently translated into a paediatric clinical setting by Emani and co-workers, who reported improved ventricular function in neonates with hypoplastic left heart syndrome following intraoperative autologous mitochondrial transplantation representing the first documented human application of therapeutic mitochondrial transfer [123]. A subsequent cohort from the same institution confirmed safety and feasibility and observed a trend toward improved cardiac function [124]. Mechanistically, transferred mitochondria are rapidly internalised by cardiomyocytes, where they restore ATP synthesis and attenuate apoptotic signalling [120,122]. In the context of ASCs specifically, tunnelling nanotube-mediated mitochondrial donation from ASCs to injured cardiomyocytes has been demonstrated in vitro, improving cardiomyocyte viability and contractility under hypoxic conditions [125]. These data raise the possibility that ASC-derived mitochondria may complement direct mitochondrial transplantation strategies in ischaemic cardiac injury, and the cardiac field’s clinical experience provides a translational roadmap that reproductive medicine can draw upon.

#### 6.7.2. Neurological Applications

Mitochondrial dysfunction is a consistent pathophysiological feature of acute brain injury and neurodegenerative disease. Hayakawa and colleagues demonstrated that astrocytes release functional mitochondria in response to neuronal injury via a CD38/cyclic ADP-ribose-dependent mechanism, and that extracellular mitochondria are internalised by neurons to rescue mitochondrial function and reduce infarct volume in a murine stroke model a discovery that fundamentally reframed our understanding of intercellular mitochondrial transfer in the CNS [126]. Subsequent in vitro studies demonstrated that ASC-derived mitochondria can be taken up by neurons under conditions of oxidative stress, with measurable improvements in respiratory chain activity and neuronal survival in models of Parkinson’s disease-relevant cytotoxicity [127]. In models of traumatic brain injury, mitochondrial transplantation into the injury zone has been shown to reduce neuronal death and improve motor recovery compared with vehicle-treated controls [128]. While these findings require substantially more evidence before clinical translation in neurology, they illustrate the breadth of the mitochondrial transplantation paradigm and suggest that the stem cell mitochondrial platform validated in reproductive biology may have neurological applications.

#### 6.7.3. Musculoskeletal and Metabolic Applications

Satellite cell mitochondrial dysfunction underpins the progressive muscle wasting observed in conditions such as Duchenne muscular dystrophy and age-related sarcopenia. Preclinical studies have demonstrated that exogenous mitochondrial supplementation improves the myogenic capacity of satellite cells and reduces oxidative stress markers in dystrophic muscle models [129,130]. ASCs have been explored as a mitochondrial donor source in this context owing to their mesenchymal lineage proximity and established paracrine support capacity. In orthopaedic contexts, mitochondrial dysfunction in chondrocytes is increasingly recognised as a driver of osteoarthritis pathogenesis; whether ASC-derived mitochondrial supplementation could support chondrocyte recovery remains an active avenue of investigation. In metabolic medicine, impaired mitochondrial function in adipocytes and hepatocytes contributes to insulin resistance and non-alcoholic fatty liver disease. ASC transplantation has been shown to improve metabolic parameters in animal models of obesity and type 2 diabetes, partly through paracrine mechanisms and partly through direct mitochondrial donation [131,132]. This finding may have particular relevance to the oocyte mitochondrial deficits observed in obese ART patients, representing a mechanistic link between systemic and reproductive metabolic dysfunction.

#### 6.7.4. Wound Healing and Dermal Regeneration

Chronic wounds particularly in diabetic patients are characterised by impaired cellular energetics, elevated oxidative stress, and mitochondrial fragmentation in wound-bed cells. ASC transplantation has consistently demonstrated benefit in preclinical wound healing models, reducing inflammatory signalling and improving re-epithelialisation rates [133,134]. Emerging evidence suggests that the mitochondrial donation capacity of ASCs, in addition to their well-established paracrine secretory activity, contributes meaningfully to these benefits: isolated ASC-derived mitochondria applied directly to wound-bed keratinocytes improved cellular viability and migration in in vitro wound-healing assays [135]. The wound healing applications of ASC-derived mitochondria are thus both independent from and conceptually related to their reproductive medicine applications, sharing the same principle of energetic rescue in metabolically compromised target cells.

#### 6.7.5. Recognized Limitations and Cautions

Despite encouraging signals across multiple organ systems, important limitations of mitochondrial transplantation must be acknowledged candidly. First, isolated mitochondria are time-sensitive: their functional integrity degrades within hours of isolation, imposing strict logistical constraints on preparation, quality assessment, and delivery [92,136]. Second, while autologous transfer is immunologically favourable, even autologous mitochondria introduced into a foreign cytoplasm may trigger innate immune responses through mitochondrial damage-associated molecular patterns (DAMPs), including mitochondrial DNA and N-formyl peptides, particularly at supraphysiological doses a concern that warrants systematic dose-finding in each application context [99]. Third, the mechanisms by which transplanted mitochondria integrate into recipient cell energy networks whether through direct fusion, biogenesis stimulation, or complementation of mtDNA defects remain incompletely understood across different cell types, complicating rational optimisation. Fourth, the persistence of transplanted mitochondria over time has not been systematically characterised across tissue types, with implications for durability of benefit. Fifth, as with any cell-derived therapy, rare mtDNA variants present in the donor ASC population could theoretically be co-transferred to recipient cells, even in autologous settings, if quality screening of the preparation is inadequate a consideration that underscores the importance of robust pre-use quality assessment. Finally, the scalability of current mitochondrial isolation methods for multi-site clinical use across diverse tissue applications remains a logistical challenge requiring standardisation.

These limitations do not diminish the promise of the mitochondrial transplantation platform but emphasise the necessity of rigorous preclinical dose-finding, mechanism elucidation, and safety evaluation before any application is considered for broad clinical adoption in reproductive medicine or elsewhere. Recognising where the evidence is strong and where it remains immature is precisely what distinguishes a measured scientific review from an advocacy document.

## 7. Conclusions

The evidence reviewed in this paper traces a coherent and compelling arc from fundamental mitochondrial biology to the threshold of clinical translation. Mitochondria are not peripheral contributors to oocyte function but are its central regulators governing the energy supply that drives meiotic maturation and early embryogenesis, the calcium dynamics that determine chromosomal fidelity, and the redox balance that protects the oocyte’s genetic material across decades of follicular dormancy. The consequences of mitochondrial dysfunction in oocytes are correspondingly far-reaching: impaired maturation, fertilization failure, embryonic arrest, aneuploidy, recurrent implantation failure, and age-related reproductive decline. These outcomes collectively define the most challenging and therapeutically unmet clinical frontier in contemporary ART.

Chemical interventions targeting mitochondrial function including CoQ10, melatonin, resveratrol, L-carnitine, and NAD^+^ precursors offer meaningful but inherently limited support for oocyte mitochondrial health. They can protect and optimize existing mitochondria but cannot replace a depleted mitochondrial pool. Heterologous mitochondrial replacement therapies achieve direct mitochondrial substitution but carry ethical, legal, and biological constraints principally the introduction of donor mtDNA, the creation of heritable germline modification, and restricted regulatory approval that confine their application to the prevention of inherited mitochondrial disease. Somatic cell mitochondrial transfer, while autologous in principle, carries unacceptable risk of transmitting accumulated mtDNA mutations to offspring and has not demonstrated consistent clinical benefit.

Against this landscape, autologous ASC-derived mitochondrial transplantation ASCENT emerges as a scientifically substantiated therapeutic strategy with several practical advantages over currently evaluated alternatives. It combines the biological advantages of a high-quality, morphologically compatible, readily accessible mitochondrial source with the ethical and regulatory simplicity of a fully autologous intervention that introduces no donor genetic material and creates no heritable genomic alteration. The preclinical evidence base for ASCENT is the most comprehensive assembled for any autologous MTT approach: functional improvement in both aged and cryopreserved oocyte models, mechanistic evidence of enhanced ATP production without increased oxidative stress, and most distinctively confirmed absence of adverse effects across three consecutive generations of offspring. The convergence of this preclinical evidence with human proof-of-concept through autologous Mito-ICSI in a clinical reproductive setting creates an unusually robust foundation for the next step in ASCENT’s development.

That next step is a rigorously designed, prospective, international clinical trial. The challenges that must be addressed in this translation standardization of mitochondrial isolation and quality assessment, determination of optimal dosing, elucidation of mechanism, definition of patient selection criteria, and establishment of appropriate regulatory and ethical governance are substantial but tractable. They are precisely the challenges that well-designed clinical research, conducted through collaborative international networks with long-term follow-up provisions, is equipped to address.

Mitochondrial medicine is reshaping our understanding of reproductive aging and infertility, and the tools to intervene directly and safely at the mitochondrial level are now within reach. ASCENT represents a meaningful milestone not because all questions have been answered, but because foundational evidence has been generated, key limitations of competing approaches are now well characterized, and a clear path to clinical translation exists. Advancing this path with scientific rigor, openness to comparative evaluation, and genuine commitment to patients represents a high priority for the field of mitochondrial therapeutics in ART. It is equally important to acknowledge that the field of autologous mitochondrial transplantation is active and evolving: iPSC-derived and endometrial stem cell-derived approaches show strong preclinical results, and any definitive ranking of approaches will ultimately be determined by head-to-head comparative clinical data rather than preclinical proxies alone.

## Figures and Tables

**Table 1:** Comparison of autologous stem cell mitochondrial transplantation approaches for oocyte quality improvement. ASC, adipose-derived stem cell; ASCENT, Adipose Stem Cell-derived Mitochondria ENergy Transfer; iPSC, induced pluripotent stem cell; Mito-ICSI, mitochondrial transfer with intracytoplasmic sperm injection; MSC, mesenchymal stem cell.

Source	Accessibility	Preclinical Evidence	Clinical Evidence	Transgenerational Safety	Key Limitation
**Oogonial stem cells**	Invasive (ovarian biopsy)	Positive	Inconsistent	Not reported	Contested cell existence; high cost
**Bone marrow MSC**	Invasive (marrow harvest)	Limited	Single series	Not reported	Invasiveness; pain
**Endometrial MSC**	Moderately invasive	Positive (aged mice)	None	Not reported	Separate cycle required
**Umbilical cord MSC**	Not autologous in adults	Positive	None	Not reported	Not applicable autologously
**iPSC**	Complex reprogramming	Strong (aged mice)	None	Not reported	Biosafety; regulatory complexity
**ASC (ASCENT)**	Minimally invasive	Strong (aged + cryo oocytes)	Proof-of-concept (Mito-ICSI)	Confirmed (3 generations)	Requires clinical trial validation

**Table 3:** Proposed multi-parametric release-criteria panel for clinical-grade mitochondrial preparations. The closest clinical precedent, human islet transplantation, applies a limit of ≤5 EU/kg recipient body weight [99]; this derives from systemic intraportal infusion and is not directly applicable to intracytoplasmic delivery of picolitre volumes. ASC, adipose-derived stem cell; EU, endotoxin unit; JC-1, 5,5′,6,6′-tetrachloro-1,1′,3,3′-tetraethylbenzimidazolylcarbocyanine iodide; LAL, Limulus amoebocyte lysate; LDH, lactate dehydrogenase; mtDNA, mitochondrial DNA; qPCR, quantitative polymerase chain reaction; RCR, respiratory control ratio; TMRM, tetramethylrhodamine methyl ester.

Parameter	Assay	Proposed Acceptance Basis	Principal Limitation
**Membrane potential (Δψm)**	JC-1 or TMRM, flow cytometry [98]	Relative to validated fresh reference preparation	Confounded by transient isolation depolarisation; not interpretable alone
**mtDNA copy number**	Duplex qPCR against single-copy nuclear gene [100]	Absolute copies per injected volume; confirms absence of nuclear DNA carryover	Sensitive to extraction method; requires locked pre-analytical protocol
**Respiratory capacity**	Extracellular flux analysis or Clark electrode [94,96]	RCR threshold, to be established for ASC-derived isolates	Reported RCR values are tissue- and protocol-specific
**Oxidative status**	Amplex Red (H_2_O_2_) or MitoSOX	Below ceiling of validated reference preparation	No threshold validated against embryo developmental outcome
**Endotoxin**	LAL assay, pharmacopoeial method	Absolute concentration (EU/mL of final suspension)	Per-kilogram systemic limits do not translate to microinjection scale
**Purity/debris**	Histone H3 and LDH immunoassay; electron microscopy [93]	Below assay detection limit	Contaminant profile depends on isolation method (Table 2)

**Table 4:** Cross-species dose–effect reference directory for autologous mitochondrial transplantation. Doses are given as reported in the source publication and are not interconvertible (see text). ASC, adipose-derived stem cell; BCB, brilliant cresyl blue; eMSC, endometrial mesenchymal stem cell; GV, germinal vesicle; ICSI, intracytoplasmic sperm injection; iPSC, induced pluripotent stem cell; MII, metaphase II; mtDNA, mitochondrial DNA; OSC, oogonial stem cell; ROS, reactive oxygen species. Studies using oogonial stem cell-derived preparations are indicated as such; outcomes from these studies should not be interpreted as pertaining to adipose-derived preparations.

Model	Study	Source	Dose as Reported	Principal Outcome
**Mouse, aged**	Wang 2017 [85]	ASC	7–8 pL (GV); 10 pL (MII, with ICSI)	mtDNA 8.4 → 12.5 × 10^4^ per MII oocyte; improved spindle integrity, reduced aneuploidy
**Mouse, aged (aged donor)**	Sheng 2019 [103]	ASC	2 pL; ~1–3% of oocyte volume	No improvement in oocyte quality
**Mouse, aged**	Zhang 2023 [79]	eMSC	7–8 pL	Improved oocyte quality
**Mouse, aged (IVF embryos)**	Zhang 2021 [80]	iPSC	5–10 pL	Rescued embryo development
**Mouse, cryopreserved oocytes**	Kankanam Gamage 2022 [16]	ASC	2–4 pL	Increased ATP without increased ROS
**Pig, mtDNA-deficient (BCB^−^) oocytes**	Cagnone 2016 [104]	Autologous oocyte-derived	3 pL; 787.5 ± 409.3 mtDNA copies per oocyte	Blastocyst rate 31.5% vs. 7.6% (IVF); 4.4-fold higher mtDNA at 2-cell stage; no benefit in mtDNA-replete oocytes
**Human, recurrent IVF failure (randomised)**	Labarta 2019 [89]	OSC	1–2 pL; ~500 mitochondria per oocyte	No difference in euploidy (*p* = 0.541); blastocyst rate 23.3% vs. 41.1%
**Human, multiple IVF failure**	Oktay 2015 [90]	OSC	2 pL	Improved outcomes; uncontrolled series
**Human, recurrent failure**	Morimoto 2023 [17]	OSC	2 pL	Improved embryo quality and live birth

## Data Availability

No new data were created or analyzed in this study. Data sharing is not applicable to this article.
